# Profiles of eHealth Adoption in Persons with Multiple Sclerosis and Their Caregivers

**DOI:** 10.3390/brainsci11081087

**Published:** 2021-08-19

**Authors:** Rocco Haase, Isabel Voigt, Maria Scholz, Hannes Schlieter, Martin Benedict, Marcel Susky, Anja Dillenseger, Tjalf Ziemssen

**Affiliations:** 1 Center of Clinical Neuroscience, Department of Neurology, University Hospital Carl Gustav Carus, Technical University of Dresden, 01307 Dresden, Germany; isabel.voigt@ukdd.de (I.V.); maria.scholz@uniklinikum-dresden.de (M.S.); anja.dillenseger@ukdd.de (A.D.); Tjalf.Ziemssen@uniklinikum-dresden.de (T.Z.); 2 Faculty of Business and Economics, Technical University of Dresden, 01062 Dresden, Germany; hannes.schlieter@tu-dresden.de (H.S.); martin.benedict@gmail.com (M.B.); marcel.susky@tu-dresden.de (M.S.)

**Keywords:** multiple sclerosis, eHealth, patient empowerment, health information seeking, user-centered design, patient portal

## Abstract

(1) Background: Persons with multiple sclerosis (pwMS) are often characterized as ideal adopters of new digital healthcare trends, but it is worth thinking about whether and which pwMS will be targeted and served by a particular eHealth service like a patient portal. With our study, we wanted to explore needs and barriers for subgroups of pwMS and their caregivers when interacting with eHealth services in care and daily living. (2) Methods: This study comprises results from two surveys: one collecting data from pwMS and their relatives (as informal caregivers) and another one providing information on the opinions and attitudes of healthcare professionals (HCPs). Data were analyzed descriptively and via generalized linear models. (3) Results: 185 pwMS, 25 informal caregivers, and 24 HCPs in the field of MS participated. Nine out of ten pwMS used information technology on a daily base. Individual impairments like in vision and cognition resulted in individual needs like the desire to actively monitor their disease course or communicate with their physician in person. HCPs reported that a complete medication overview, additional medication information, overview of future visits and a reminder of medication intake would be very helpful eHealth features for pwMS, while they themselves preferred features organizing and enriching future visits. (4) Conclusions: A closer look at the various profiles of eHealth adoption in pwMS and their caregivers indicated that there is a broad and robust enthusiasm across several subgroups that does not exclude anyone in general, but constitutes specific areas of interest. For pwMS, the focus was on eHealth services that connect previously collected information and make them easily accessible and understandable.

## 1. Introduction

Persons with multiple sclerosis (pwMS) are often characterized as early or ideal adopters of new digital healthcare trends [1]. They are faced with a disease that usually starts in early adulthood and warrants the attention of pwMS, their relatives, and several types of healthcare professionals (HCPs) for the rest of patients’ life. The disease itself is complex and may result in a multitude of different symptoms [2,3]. Overall, every person involved has to put a lot of effort in treatment management and associated multimodal monitoring, which will generate large numbers of multidimensional data [4]. Modern standardized disease management of multiple sclerosis (MS) should therefore include time- and cost-saving health information technology (HIT) that improves the conditions for and the connections between pwMS, HCPs, and informal caregivers [5,6].

We know from cross-domain research that younger patients and patients who are more active IT users are particularly interested in and have established skills with using eHealth services [7,8]. With the understanding that these characteristics are connected to pwMS [1], research on eHealth in the domain of MS has focused on creating a variety of new technologies to aid in the diagnostic and monitoring process and treatment of MS [6,9,10]. One focus is on providing new methods for networked generation of data, particularly to address symptom domains underrepresented in the standard MS assessment and temporal gaps in data collection. These approaches include self-monitoring via mobile health technologies, new (wearable) sensor systems like accelerometers, as well as extended and adapted electronic health records (EHR). All these efforts are aimed at preventing the disease from progressing unnoticed and unanswered [11].

A second major area of research on eHealth in MS deals with sharing and retrieving health-related information and experiences. In recent years, the availability of high-quality information about MS for pwMS and the ways to disseminate this knowledge have increased steadily [9]. Research on patient networks, professionally curated websites, and how patients find and process information online have gained much attention [9,12,13,14,15].

The goal of almost all of these eHealth services was to address all pwMS in a similar manner, or at least all within a given language area, which maintains the assumption that all pwMS are potential eHealth adopters, or at least that they all are to a similar degree. The low age at onset [16] and the general trend toward more digital devices [17] may suggest this conclusion, but it is also contradicted by the wide range of possible symptoms, the growing age of patients under treatment, the large number of HCPs involved, and the dispersion of those affected by MS within a society [5,16]. However, there is evidence that it is worth thinking about whether and which pwMS will be targeted and served by a particular eHealth service [15,18,19,20].

User-centered design (UCD) in the development of eHealth services represents an important principle for the differentiated consideration of attitudes and wishes of designated users like patients and HCPs [21,22]. For a development according to UCD, the following questions arise. Are all potential users identified as both information sources and target users? What are key needs, barriers and success factors for these individuals? Are differentiating factors such as a priori usage behavior, socio-demographic, and motivational characteristics considered? Are quantitative and qualitative methods used to capture outcomes and factors?

In a mixed-method study of Giunti et al., twelve pwMS and twelve HCPs from Switzerland were interviewed and assessed with questionnaires to gain insights into the needs of pwMS when using a mobile app to increase physical activity [22]. While pwMS and HCPs were used as a source of information in this regard, HCPs were not seen as active partners in the process of optimizing patients’ physical activity through an app. As Giunti et al. themselves noted, the results of the study were based on a relatively small number of cases and a prelimited study population, which significantly reduced overall representativeness and did not allow for systematic multifactorial analyses. Marrie et al. contributed a very large study on usage behavior of pwMS in North America, which also systematically looked for differences in subgroups of pwMS. With more than 6400 pwMS, a very broad data set was created and numerous important factors were used in multivariate analyses, but the outcomes collected were often only binary and mostly addressed the general IT use of the pwMS. This is due to the epidemiological approach, which, unlike the UCD-based development process of a specific application, does not target a specific application purpose and thus provides less detail, especially for attitudes and needs.

In our study, we aimed to combine the strengths of these two approaches by asking detailed questions about current and potential usage patterns of eHealth services for MS and linking them to various factors so that systematic associations with subgroups are revealed. Since this study was designed in the context of a UCD-based software development, many involved perspectives should be considered both as information providers and as participants in the usage process. Specifically, this study sought to explore how willing pwMS (and their caregivers) would be to interact with their electronic health record, use alternative communication channels, and incorporate mobile devices and eHealth apps into care and their daily lives.

## 2. Materials and Methods

The research was conducted as part of the joint project “Integrated Care Portal for Multiple Sclerosis” by the Multiple Sclerosis Center (MSC) at University Hospital Carl Gustav Carus Dresden, the Technical University of Dresden (TUD), Chair of Wirtschaftsinformatik, Systems Engineering, and the Carus Consilium Sachsen GmbH between October 2017 and November 2019 [23]. This study comprises results from two surveys that were created by a team of neurologists, psychologists, and computer scientists at the MSC and TUD to enable a user-entered development process for an integrative care portal for MS including the perspectives of the most important persons involved.

The first survey collected data from pwMS and their relatives (as informal caregivers) and the second survey provided information on the opinions and attitudes of the neurologists treating MS. In both surveys, we gathered information concerning the use of information technology, disease-related barriers, requirements and needs for adopting eHealth solutions for MS.

### 2.1. Participants

PwMS and their relatives were enrolled during routine visits at the MSC and events like an information day and via support groups of the German Multiple Sclerosis Society. Questionnaires should only be given to persons with a verified diagnosis of MS and their relatives. No further restrictions were made to include a representative range of patients. The paper-based questionnaire could be filled in during the visit/event or later be submitted at the next appointment, by post or electronically by email as a scan. All submitted questionnaires were processed anonymously.

HCPs from Germany treating MS were contacted by mail and email through a network list of neurological practices to answer questions of the second survey anonymously online via web link. Experts must have treated pwMS regularly and have at least two years of personal experience in the field to provide appropriate answers for the analyses, which was ensured with initial items on these aspects.

### 2.2. Data Collection

#### 2.2.1. Patient Survey

The questionnaire assessing pwMS and their relatives consisted of 65 items including information about their background and their condition as well as their attitudes and behavior regarding the eHealth in the context of MS. To include relevant patient characteristics in the analyses, we asked the patients with ordinal and bivariate items about their age, the distance to their treating neurologist, whether they suffered from symptoms of fatigue, depression, pain, spasticity, impaired cognition, walking ability, vision, bladder and bowel function, or other symptoms due to their MS. We added up the presence of these symptoms to estimate a severity index ranging from 0 to 10 for further analyses. Further questions concerned the frequency and purpose of use of digital devices and reasons for not using such tools for health-related tasks, especially to manage their MS. Answers were provided on a 5-point Likert scale or as dichotomous outcome. The second part of this survey focused on expectations and needs regarding the use of a common eHealth infrastructure that is accessible for pwMS and their caregivers as well.

#### 2.2.2. Physician Survey

With a second survey for HCPs in the field of MS, we wanted to address their attitudes towards eHealth for treating MS, their current use of HIT and their needs and requirements for a an eHealth environment that connects pwMS as well as their respective formal and informal caregivers. In total, 100 items were used to assess HCPs basic characteristics (8 items), their current way of using HIT (13 items), information about processes in the clinical practice (32 items), needs and opinions about a patient portal for MS (40 items), and electronic health records for MS (7 items). Responses were given as free text, 5-point Likert scale or multiple-choice option. HCPs could skip items if they did not feel qualified to answer.

### 2.3. Statistical Analyses

Absolute and relative numbers, median and interquartile range were used to describe the study variables. Percentages based only on complete answers. When recoding of ordinal data was required, the two most favorable outcomes on a 5-point Likert scale were coded as favorable binary outcome. For a deeper understanding of the patterns in which subgroups in both surveys may differ, generalized linear models (GLMs) with binomial and multinomial link function were applied to analyze responses with respect to individual characteristics. A *p*-value < 0.05 was considered significant. For analyses of the patient survey, the model factors included age, type of participant, distance to and type of treating neurologist, disease severity (via index), and major symptom classes (fatigue, depression, cognition, pain, spasticity, walk, vision, bladder, bowel). Wilcoxon tests were used to compare ordinal ratings. Kendall’s tau–b (τ) was used to estimate agreement for ordinal ratings.

## 3. Results

### 3.1. Participants

Overall, 185 pwMS, 25 informal caregivers, and 24 healthcare professionals in the field of MS participated in our surveys.

In the first survey, the age of pwMS and their relatives ranged from 18 to over 60 years with a median of 31 to 40 years. Most cases were treated at the MSC (74.3%) while 25.7% were treated in general neurological practices and clinics. The median distance pwMS had to travel to treat HCP was between 5 to 15 km. Major self-reported symptoms were fatigue (58.6), impairment of walking (51.0%), vision (35.2%), cognition (31.9%), as well as pain (31.9%), bladder problems (28.6%), other symptoms (22.4%), depression (20.5%), and bowel problems (11.9%) with an average of 3.14 symptoms per patient.

In the second survey, 20 neurologists, 2 radiologists, and 2 specialized MS nurses answered our questions with a median experience of 11 to 25 years in the field of MS. Participating HCPs working in neurological practices (50%) and clinics (41.7%) treated an average of 901 patients per quarter with 15.5% of these patients being pwMS (range between 1% and 100%).

### 3.2. Patient Survey

Overall, 89.0% of pwMS and their caring relatives used information technology on a daily base (Table 1). Only 5.7% of them did not use or rarely used devices such as a smartphone or a computer with the smartphone being the most used device.

Typical health-related tasks that include the use of HIT were accessing health information on the Internet and self-tracking (Table 2). Finding a new physician and contacting physicians were the least common use cases for our subjects. The most common reasons for not using HIT solutions were the lack of knowledge about existing services (17.6%), concerns about the usefulness of a service (11.4%), low familiarity with the technology (9.5%), a lack of trust in existing services (5.7%), and other reasons (21.9%).

When being asked whether they had ever accessed information about MS from a specific source, 76.2% of the participants answered that they had used the Internet, 75.2% contacted a specialized physician, 41.0% read books and magazines, 21.4% visited MS-related events, 20.5% talked to other patients, 2.9% used an app for MS, and 7.6% accessed other sources. Barriers for accessing information about MS were the general lack of understandability (76.3%) as well as the accessing (53.4%), overviewing (51.0%), and understanding (38.7%) of personal health records and the unavailability of suitable information (13.9%) and other patients to communicate with (43.0%). A patient portal for MS was welcomed by 93.1% of the participants. At the MS Day event of the MSC in 2019, we also asked our attending pwMS whether they would be willing to pay for the use of such a portal (*N* = 60) and 61.7% of them were willing to do so in principle. Most desired features of such a portal were the access to personal EHRs (85.9%), an overview of the medication schedule (82.6%), additional information about the current treatment (86.9%), and an overview of past (74.0%) and future visits (87.3%). Also of interest to some degree were the options to remind patients for taking their medication (57.7%), as well as to mail (81.1%), call (49.2%), and video chat (35.3%) with physicians.

In analyses with GLMs, we found several significant associations between distinct subject characteristics and their behaviors and attitudes toward eHealth for MS (Table 3).

Individual impairments like in vision and cognition resulted in individual interests like the desire to actively monitor their disease course or communicate with their physician in person. As expected, pwMS were more interested in actively managing their disease than their informal caregivers who were nevertheless interested in many aspects of the disease. Participants who were associated to a highly specialized MS care unit like the MSC showed an increased interest in interactive possibilities of eHealth for MS like the possibility to do tests and questionnaires online and via a mobile accessible platform like a patient portal for pwMS. Also as expected, younger participants presented with an increased frequency of using modern communication devices. The general interest in a patient portal for MS did not differ between subgroups.

### 3.3. Physician Survey

All HCPs had already used HIT-like specific software on computers (100%) and mobile devices (65%) in their practices and clinics. Only a minority of them processed imaging data via the Internet (31.8%), which is a typical use case for diagnostics in MS. Another 65% already provided additional educational programs to their patients. Median number of contacts between HCPs and their patients was up to two times per quarter. Among common problems that HCPs were facing during disease management, the lack of forwarding of information by the patient (31.6%), the need for the patient to visit on site for inquiries (21.1%), a missing overview of treatments including those from other HCPs, and poor general reachability of patients (15.8%) were the most prominent ones.

Seen from the HCPs’ point of view, a complete medication overview, additional medication information, overview of future visits, and a reminder of medication intake would be very helpful portal features for pwMS (Table 4, Appendix A). Helpful portal features for HCPs themselves were medication overview, overview of future visits, and preparing appointments so that pwMS know what to expect and what to bring with them. For most of the tasks of a common online portal for MS, a trend towards having more benefits for patients was observed, but only the contact via text message was rated significantly more favorable for pwMS (*p* = 0.016) than for HCPs. No differences were found for the ratings with respect to the HCPs’ characteristics like working in clinics vs. in practices, their occupation (neurologist, radiologist, MS nurse), their professional experience, and or the share of pwMS among all treated patients.

The highest level of agreement for perceived use rated for patients and physicians was found in the systematic overview of past visits (τ = 0.882), followed by video chats (τ = 0.825), reminders for patients (τ = 0.786), and medical overview (τ = 0.713). The only non-significant correlation was detected for doing questionnaires and tests via patient portal (τ = 0.235).

## 4. Discussion

In our study, we assessed the current and potential use of HIT by pwMS as well as by their formal and informal caregivers from a unified perspective and connected their answers with disease and treatment specific characteristics to promote a more detailed view on different profiles of eHealth adoption in MS.

We found that it was of particular importance for pwMS to get an effective access to their own medical data, especially treatment-related and visit-related data. This perspective was shared with HCPs treating MS. While there were high levels of use of modern communication technologies among all participating groups, we were able to identify significant differences in usage patterns as well as needs and experienced barriers to the use of eHealth technologies for MS.

As expected, younger pwMS were more receptive to modern communication technologies, but also pwMS and their relatives who had already experienced additional eHealth services in routine practice were more open towards the possibilities of a complex solution like an integrative patient portal for MS [6]. PwMS and their relatives shared many attitudes and knowledge about the disease and its treatment, but pwMS themselves were more interested in actively supporting disease management through electronically aided visit management. Patients with specific problems such as cognitive functional deficits were more interested in options to cope with these symptoms via a mobile-available assessment.

Research on the use of HIT in MS has evolved in the last ten years. In a number of studies, device use patterns and online search behavior for health information in pwMS [1,14,15,24,25] were assessed in several countries around the world. Our current study followed that tradition and updated previously established numbers with actual insights from a society that adopted a widespread use of smartphones across all subgroups [1]. For pwMS, we found that nine out of ten patients could be reached through modern communication devices, which extends the trend of previous studies [19,26]. Our numbers correspond to 90% of people in the general German population using a smartphone in 2020 [27]. Daily and weekly usage of HIT-ready devices and the Internet in general were the desired levels at which responsive disease management could be started [28]. For many routine tasks, access of eHealth services on a weekly or monthly base seemed sufficient for our pwMS, for example, to contact caregivers or to receive new information on MS. Therefore, a very high frequency in the use of HIT was not necessary for a successful adoption of eHealth services. Mobile applications that offer self-tracking and optimization options such as physical or cognitive trainings may be seen as one option that justifies a more frequent use of web-based services for MS [11,29].

Another approach that we wanted to take with this study is the multi-perspective research on needs and barriers that pwMS and caregivers face while managing MS, and that should be met and overcome by technical solutions [18,22]. To achieve this, patients and caregivers should be understood both as a source of information and as a target group for the development of eHealth solutions, and disease management itself as an interaction between these parties to be supported. Therefore, it was necessary to gather detailed requirements that should be met by a common web portal for pwMS and their caregivers, and to investigate whether these apply equally to subgroups. There was an increased need to access their EHR in pwMS with a large number of different functional deficits. However, in pwMS with impaired vision, we found less interest in accessing their EHR online. Instead, they contacted their specialized physician more often to get MS-related information directly from them, which was also seen in pwMS that lived near their treating HCPs. Here, we also see the potential that eHealth has for the care landscape in rural areas, which generally have a lower density of HCPs. Where face-to-face contact is rarely possible, more demand for information on MS can be served online. Optimizing readability may also promote the use of eHealth apps among pwMS with visual impairments. While younger pwMS and their relatives reported a more frequent use of modern communication devices, we also noted an increased interest in eHealth solutions in pwMS having already used such applications at their treating neurologist. While we cannot influence the age of pwMS, a high-quality offer of new eHealth methods like a patient portal by the practitioner may increase openness to them.

From HCPs, we learned that eHealth services can be equally important to patients and their caregivers when both were able to access them. For a common online portal for MS, features to overcome organizational and communicational deficits were most anticipated by HCPs. The benefits of this solution may also include improved patient education and networking and data sharing with other participating HCPs. Features that had already been implemented elsewhere, such as HCP’s access to EHR and the ability to take mail and calls from patients, were met with less interest. This underlines the need for clear additional benefits for all stakeholders that should come with the use of new HIT.

In a study by Nielsen et al., pwMS that were already using an online portal focusing on patient–physician communication and accessing EHRs at the Beth Israel Deaconess Medical Center were assessed [20]. Like in our study, recommendations like font size adjustments were provided to overcome barriers related to physical disabilities. Further, younger age and normal vision were factors that predicted portal use. As this was a retrospective study, no specific questions could be answered. Atreja et al. used focus groups to get insights into needs and barriers of an Internet portal for MS [25]. Both studies have in common that they saw only patients as beneficiaries of the portal, yet envisioned HCPs using it for communication without primarily considering them in the portal design. Common recommendations from these studies and our findings include consideration of differential accessibility for patients with special impairments, integration of PROs and tests, and the objective that a patient portal must directly support shared physician–patient decision making, but certainly in ways that are different for physicians and patients.

Nevertheless, we have to address some limitations of our surveys. Since recruitment was on a voluntary basis, selection bias could have been present. The survey addressing HCPs achieved only a small sample size, which may have limited the representativeness of the findings and the power for statistical tests. In the survey directed to pwMS, we had to use binary surrogate items for assessing clinical symptoms. The use of a standard clinical instrument like the Expanded Disability Status Scale was prohibited due to the anonymous survey process [30]. Further, the use of the category “other symptoms” may have limited insights into further symptom areas like sensory impairment. A proportion of unanswered items reduced the amount of information available and may have reduced the number of responses in the “rarely or never” category, as participants may have omitted questions primarily when they did not apply to them personally.

## 5. Conclusions

Overall, pwMS as well as their formal and informal caregivers showed high interest in eHealth solutions for MS. A closer look at the various profiles of eHealth adoption indicated that there is a broad and robust enthusiasm across several subgroups of pwMS that does not exclude anyone in general, but constitutes specific areas of interest.

For pwMS, the main focus was on MS care portal options that connect previously collected information and make them easily accessible and understandable. For HCPs, organizing and enriching future visits was an important aspect.

Overall, a well-established, multilingual, standardized questionnaire on the usage behavior of modern communication devices and platforms would be a welcomed starting point for cross-domain comparable research on the topic.

With our integrated care portal and the vision of digital twins for MS, patient involvement will be strengthened by a purposeful assistance in organizing and caring [23,31]. In addition, context-sensitive information for patients and their relatives as well as concrete recommendations and options for action based on this information will be provided.

## Figures and Tables

**Table 1:** Frequencies of information technology use in persons with multiple sclerosis and their relatives (*N* = 210).

Use of Device	Parameter	Daily	Weekly	Less than Weekly	Unknown
Computer or Notebook	*n*	110	51	29	20
	%	57.9	26.8	15.3	
Tablet	*n*	61	24	47	78
	%	46.2	18.2	35.6	
Smartphone	*n*	158	3	12	37
	%	91.3	1.7	6.9	
Smartwatch	*n*	10	2	66	132
	%	12.8	2.6	84.6	
Any of These Devices	*n*	187	11	12	0
	%	89.0	5.2	5.7	

Percentages based on complete answers.

**Table 2:** Purpose of use of information technology use in persons with multiple sclerosis and their relatives (*N* = 210).

Use of Device	Parameter	Daily or Weekly	Monthly	Rarely or Never	Unknown
Access General Information on Health	*n*	59	62	54	35
	%	33.7	35.4	30.9	
Access Information on Multiple Sclerosis	*n*	45	65	70	30
	%	25.0	36.1	38.9	
Find a New Physician	*n*	12	56	102	40
	%	7.1	32.9	60.0	
Self-Tracking	*n*	22	10	113	65
	%	15.2	6.9	77.9	
Organize Appointments	*n*	44	43	77	49
	%	27.3	26.7	47.8	
Exchange with Other Patients	*n*	22	17	114	57
	%	14.4	11.1	74.5	
Contact Physicians	*n*	10	51	101	48
	%	6.2	31.5	62.3	

Percentages based on complete answers.

**Table 3:** Associations between subgroups of participants and their behaviors and attitudes toward eHealth for multiple sclerosis (MS) (*N* = 210).

Characteristic	Association	*p*
Younger Participants	Used any Modern Communication Device More Often	0.001
	Used Tablets More Often	0.028
	Used Smartphones More Often	0.031
	Looked More Often for a New Physician Online	<0.001
	Participated Less Often in Live Events about MS ^1^	0.004
	Were More Interested in an Overview of Future Visits	0.013
Participants with Lower Distance to the Treating Physician	Received More Often MS-Related Information Directly from Their Specialized Physician	0.040
Participants being Treated in a Highly Specialized MS Unit	Were More Likely to Use Modern Communication Devices for Retrieving Health Information	<0.001
	Were Less Likely to Attend Live Events about MS	0.034
	Were More Interested in an Overview of Future Visits ^1^	0.013
	Were More Interested in Filling in Questionnaires and Tests ^1^	0.030
	Were More Interested in Managing Visits ^1^	0.004
Participants with an Increased Number of MS Symptoms	Were More Interested in Accessing their Electronic Health Record ^1^	0.026
Persons with MS in Comparison with Friends and Relatives of Persons with MS	Were More Likely to Use Modern Communication Devices for Retrieving Health Information	0.006
	Were More Interested in MS-Related Reminders ^1^	0.001
	Were More Interested in an Overview of Past Visits ^1^	0.044
	Were More Interested in an Overview of Future Visits ^1^	0.012
	Were More Interested in Managing Visits ^1^	0.009
Participants with Fatigue	Used any Modern Communication Device Less Often	0.001
	Used Computers and Notebooks Less Often	0.032
	Looked More Often for a New Physician Online	0.005
Participants with Cognition Problems	Used Computers and Notebooks Less Often	0.043
	Were More Likely to Look for Information on MS Online	0.006
	Were More Interested in Filling in Questionnaires and Tests ^1^	0.047
Participants with Walking Problems	Looked Less Often for a New Physician Online	0.030
Participants with Vision Problems	Received more Often MS-Related Information Directly from their Specialized Physician	0.016
	Were Less Interested in in Accessing their Electronic Health Record ^1^	0.036

Generalized linear models were used with binomial and multinomial link function and age, type of participant, distance to and type of treating neurologist, number of symptoms, and major symptom classes (fatigue, depression, cognition, pain, spasticity, walk, vision, bladder, bowel) as factors. Only significant associations are displayed (*p* < 0.050). ^1^ Via an online portal for persons with multiple sclerosis and their caregivers.

**Table 4:** Healthcare professionals’ ratings for useful features of an online portal for persons with multiple sclerosis and their caregivers (*N* = 24).

Task	Parameter	Useful for the Patient	Useful for the Physician	Unknown	τ
Access Electronic Health Record	*n*	9	5	7	0.631 ^1^
	%	52.9	29.4		
Medical Overview	*n*	16	15	7	0.713 ^1^
	%	94.1	88.2		
Patient Inquiries	*n*	13	10	7	0.548 ^1^
	%	76.5	58.8		
Treatment Information	*n*	14	13	7	0.487 ^1^
	%	82.4	76.5		
Reminder for Treatment	*n*	13	13	7	0.786 ^1^
	%	76.5	76.5		
Overview of Past Visits	*n*	9	9	7	0.882 ^1^
	%	52.9	52.9		
Overview of Future Visits	*n*	14	14	7	0.659 ^1^
	%	82.4	82.4		
Contact via Text Message	*n*	9	4	7	0.550 ^1^
	%	52.9	23.5		
Contact via Audio Call	*n*	7	5	7	0.663 ^1^
	%	41.2	29.4		
Contact Via Video Call	*n*	6	5	7	0.825 ^1^
	%	35.3	29.4		
Questionnaires and Tests	*n*	13	12	7	0.235
	%	76.5	70.6		
Prepare Visits	*n*	12	13	7	0.652 ^1^
	%	70.6	76.5		
Post-Visit Tasks and Control	*n*	9	12	7	0.704 ^1^
	%	52.9	70.6		

Percentages and correlations based on complete answers. Kendall’s tau–b (τ) was used for correlations between rated usefulness for the patient and the physician. ^1^ Significant correlation on a at least 5% level.

## Data Availability

The data presented in this study are available on reasonable request from the corresponding author.

## Appendix A

**Table A1:** Standardized regression coefficients for significant associations in generalized linear models from Table 3 (*N* = 210).

Outcome	Factor	B1	B2	B3	B4	B5
Used of Any Modern Communication Device	Age	−1.670	−1.181	−0.485	0.298	0 ^2^
	Fatigue	−1.512	0 ^2^	–	–	–
Use of Computers and Notebooks	Fatigue	−0.817	0 ^2^	–	–	–
	Cognition	−0.879	0 ^2^	–	–	–
Use of Tablets	Age	−0.480	−1.005	0.167	0.397	0 ^2^
Use of Smartphones	Age	−0.953	−0.707	0.156	0.762	0 ^2^
Use Modern Communication Devices for Retrieving Health Information	Type of Participant	2.043	0 ^2^	–	–	–
	Being Treated in a Highly Specialized MS Unit	−2.700	0 ^2^	–	–	–
Look for a New Physician Online	Age	−2.266	−0.756	−1.156	0.233	0 ^2^
	Fatigue	−1.426	0 ^2^	–	–	–
	Walking	2.444	0 ^2^	–	–	–
Look for Information on MS Online	Cognition	1.957	0 ^2^	–	–	–
Participated in Live Events About MS ^1^	Age	2.439	2.493	1.440	0.651	0 ^2^
	Being Treated in a Highly Specialized MS Unit	0.979	0 ^2^	–	–	–
Receive Information on MS Directly from a Specialized Physician	Distance to Physician	−1.149	−0.988	0.016	0 ^2^	–
	Vision	1.371	0^2^	–	–	–
Accessing Electronic Health Records ^1^	Disease Severity	0.716	–	–	–	–
	Vision	−0.939	0 ^2^	–	–	–
Medication Reminder ^1^	Type of Participant	1.678	0 ^2^	–	–	–
	Age	−1.882	−2.127	−1.749	−0.882	0 ^2^
Overview of Past Visits ^1^	Type of Participant	1.001	0 ^2^	–	–	–
Overview of Future Visits ^1^	Type of Participant	1.488	0 ^2^	–	–	–
	Age	−1.223	−1.210	−0.843	0.090	0 ^2^
	Being Treated in a Highly Specialized MS Unit	−0.897	0 ^2^	–	–	–
Filling in Questionnaires and Tests ^1^	Being Treated in a Highly Specialized MS Unit	−0.750	0 ^2^	–	–	–
	Cognition	0.931	0 ^2^	–	–	–
Manage Visits ^1^	Type of Participant	1.437	0 ^2^	–	–	–
	Being Treated in a Highly Specialized MS Unit	−0.987	0 ^2^	–	–	–

Generalized linear models were used with binomial and multinomial link function and age, type of participant, distance to and type of treating neurologist, number of symptoms, and major symptom classes (fatigue, depression, cognition, pain, spasticity, walk, vision, bladder, bowel) as factors. Only significant associations are displayed (*p* < 0.050). ^1^ Via an online portal for persons with multiple sclerosis and their caregivers. ^2^ Reference category.

## References

[1] Haase R., Schultheiss T., Kempcke R., Thomas K., Ziemssen T. (2012). Use and Acceptance of Electronic Communication by Patients with Multiple Sclerosis: A Multicenter Questionnaire Study. J. Med. Internet Res..

[2] Ziemssen T. (2011). Symptom management in patients with multiple sclerosis. J. Neurol. Sci..

[3] Compston A., Coles A. (2008). Multiple sclerosis. Lancet.

[4] Ziemssen T., Kern R., Thomas K. (2016). Multiple sclerosis: Clinical profiling and data collection as prerequisite for personalized medicine approach. BMC Neurol..

[5] Hobart J., Bowen A., Pepper G., Crofts H., Eberhard L., Berger T., Boyko A., Boz C., Butzkueven H., Celius E.G. (2019). International consensus on quality standards for brain health-focused care in multiple sclerosis. Mult. Scler. J..

[6] Scholz M., Haase R., Schriefer D., Voigt I., Ziemssen T. (2021). Electronic Health Interventions in the Case of Multiple Sclerosis: From Theory to Practice. Brain Sci..

[7] Tennant B., Stellefson M., Dodd V., Chaney B., Chaney D., Paige S., Alber J. (2015). eHealth Literacy and Web 2.0 Health Information Seeking Behaviors Among Baby Boomers and Older Adults. J. Med. Internet Res..

[8] Neter E., Brainin E. (2012). eHealth Literacy: Extending the Digital Divide to the Realm of Health Information. J. Med. Internet Res..

[9] Lavorgna L., Brigo F., Moccia M., Leocani L., Lanzillo R., Clerico M., Abbadessa G., Schmierer K., Solaro C., Prosperini L. (2018). e-Health and multiple sclerosis: An update. Mult. Scler. J..

[10] Marziniak M., Brichetto G., Feys P., Meyding-Lamadé U., Vernon K., Meuth S.G. (2018). The Use of Digital and Remote Communication Technologies as a Tool for Multiple Sclerosis Management: Narrative Review. JMIR Rehabil. Assist. Technol..

[11] Allen-Philbey K., Middleton R., Tuite-Dalton K., Baker E., Stennett A., Albor C., Schmierer K. (2020). Can We Improve the Monitoring of People with Multiple Sclerosis Using Simple Tools, Data Sharing, and Patient Engagement?. Front. Neurol..

[12] Wicks P., Massagli M., Kulkarni A., Dastani H., Jacobs D., Jongen P. (2011). Use of an Online Community to Develop Patient-Reported Outcome Instruments: The Multiple Sclerosis Treatment Adherence Questionnaire (MS-TAQ). J. Med. Internet Res..

[13] Jongen P.J., Sinnige L.G., Van Geel B.M., Verheul F., Verhagen W.I., Van Der Kruijk R.A., Haverkamp R., Schrijver H.M., Baart J.C., Visser L.H. (2015). The interactive web-based program MSmonitor for self-management and multidisciplinary care in multiple sclerosis: Concept, content, and pilot results. Patient Prefer. Adherence.

[14] Lejbkowicz I., Paperna T., Stein N., Dishon S., Miller A. (2010). Internet Usage by Patients with Multiple Sclerosis: Implications to Participatory Medicine and Personalized Healthcare. Mult. Scler. Int..

[15] Marrie R.A., Salter A.R., Tyry T., Fox R.J., Cutter G.R. (2013). Preferred Sources of Health Information in Persons with Multiple Sclerosis: Degree of Trust and Information Sought. J. Med. Internet Res..

[16] Koch-Henriksen N., Sorensen P.S. (2010). The changing demographic pattern of multiple sclerosis epidemiology. Lancet Neurol..

[17] Pew Research Center (2021). Internet/Broadband Fact Sheet. https://www.pewresearch.org/internet/fact-sheet/internet-broadband/.

[18] Kern R., Haase R., Eisele J.C., Thomas K., Ziemssen T., Neter E., Mosconi P., Colombo C. (2016). Designing an Electronic Patient Management System for Multiple Sclerosis: Building a Next Generation Multiple Sclerosis Documentation System. Interact. J. Med. Res..

[19] Marrie R.A., Leung S., Tyry T., Cutter G.R., Fox R., Salter A. (2019). Use of eHealth and mHealth technology by persons with multiple sclerosis. Mult. Scler. Relat. Disord..

[20] Nielsen A.S., Halamka J.D., Kinkel R.P. (2012). Internet portal use in an academic multiple sclerosis center. J. Am. Med. Inform. Assoc..

[21] Dabbs A.D.V., Myers B.A., MC Curry K.R., Dunbar-Jacob J., Hawkins R.P., Begey A., Dew M.A. (2009). User-Centered Design and Interactive Health Technologies for Patients. Comput. Inform. Nurs..

[22] Giunti G., Kool J., Romero O.R., Zubiete E.D., Salcedo V.T., Jongerius C., Lyons E., Corbett T. (2018). Exploring the Specific Needs of Persons with Multiple Sclerosis for mHealth Solutions for Physical Activity: Mixed-Methods Study. JMIR mHealth uHealth.

[23] Voigt I., Benedict M., Susky M., Scheplitz T., Frankowitz S., Kern R., Müller O., Schlieter H., Ziemssen T. (2020). A Digital Patient Portal for Patients with Multiple Sclerosis. Front. Neurol..

[24] Hay M.C., Strathmann C., Lieber E., Wick K., Giesser B. (2008). Why patients go online: Multiple sclerosis, the internet, and physician-patient communication. Neurologist.

[25] Atreja A., Mehta N., Miller D., Moore S.M., Nichols K., Miller H., Harris C.M. (2005). One size does not fit all: Using qualitative methods to inform the development of an Internet portal for multiple sclerosis patients. AMIA Annu. Symp. Proc..

[26] Haase R., Schultheiss T., Kempcke R., Thomas K., Ziemssen T. (2013). Modern communication technology skills of patients with multiple sclerosis. Mult. Scler. J..

[27] Statista (2021). Persönliche Gerätenutzung für den Medienkonsum in Deutschland in den Jahren 2014 bis 2020. https://de.statista.com/statistik/daten/studie/476467/umfrage/persoenliche-geraetenutzung-fuer-den-medienkonsum-in-deutschland/.

[28] Potemkowski A., Brola W., Ratajczak A., Ratajczak M., Zaborski J., Jasińska E., Pokryszko-Dragan A., Gruszka E., Dubik-Jezierzańska M., Podlecka-Piętowska A. (2019). Internet Usage by Polish Patients with Multiple Sclerosis: A Multicenter Questionnaire Study. Interact. J. Med. Res..

[29] Giunti G., Rivera-Romero O., Kool J., Bansi J., Sevillano J.L., Granja-Dominguez A., Izquierdo-Ayuso G., Giunta D. (2020). Evaluation of More Stamina, a Mobile App for Fatigue Management in Persons with Multiple Sclerosis: Protocol for a Feasibility, Acceptability, and Usability Study. JMIR Res. Protoc..

[30] Kurtzke J.F. (1983). Rating neurologic impairment in multiple sclerosis: An expanded disability status scale (EDSS). Neurology.

[31] Voigt I., Inojosa H., Dillenseger A., Haase R., Akgün K., Ziemssen T. (2021). Digital Twins for Multiple Sclerosis. Front. Immunol..

